# The effect of price on cigarette consumption, distribution, and sale in Tehran: a qualitative study

**DOI:** 10.1186/s12889-021-11733-5

**Published:** 2021-09-22

**Authors:** Younes Panahi Golestan, Mohammad Ebrahimi Kalan, Ziyad Ben Taleb, Kenneth D. Ward, Mehdi Fazlzadeh, Raed Bahelah, Mohammad Reza Masjedi, Abdurrahman Charkazi, Nasir Dehghan, Shirin Shahbazi Sighaldeh

**Affiliations:** 1 Department of Social science, Farhangian University, Karaj, Iran; 2 Department of Epidemiology, Robert Stempel College of Public Health, Florida International University, Miami, FL USA; 3 Department of Kinesiology, College of Nursing and Health Innovation, University of Texas at Arlington, Arlington, TX USA; 4 School of Public Health, University of Memphis, Memphis, TN USA; 5 Department of Environmental Health, School of Health, Ardabil University of Medical Sciences, Ardabil, Iran; 6 School of Health Sciences, Baldwin Wallace University, Berea, OH USA; 7 Department of pulmonary medicine, Shahid Beheshti University of Medical Sciences, Tehran, Iran; 8 Cancer Control Research Center, Cancer Control Foundation, Iran University of Medical Sciences, Tehran, Iran; 9 Tobacco Control Research Center (TCRC), Iranian anti-tobacco association, Iran University of Medical Sciences, Tehran, Iran; 10 Health Education and Promotion, Environmental Health Research Center, Golestan University of Medical Sciences, Golestan, Gorgan, Iran; 11 Preventive Medicine and Public Health Research Center, Iran University of Medical Sciences, Tehran, Iran; 12 Department of Midwifery and Reproductive Health, School of Nursing and Midwifery, Tehran University of Medical Sciences, Tehran, Iran; 13 Nursing and Midwifery Care Research Center, Tehran University of Medical Sciences, Tehran, Iran; 14 Breastfeeding research center, Tehran University of Medical Sciences, Tehran, Iran

**Keywords:** Cigarette sale, Cigarette tax, Smuggling, Cigarette distribution, Qualitative study

## Abstract

**Background:**

Appropriate increases in tobacco taxes and prices are an essential component of comprehensive tobacco control strategies. This study investigates factors related to the use, sale, and distribution of cigarettes in Iran, focusing on the relationship between cigarette price and its consumption.

**Methods:**

This interview-based qualitative study was conducted among 20 participants, including cigarette smokers, retail shop owners, large-scale distributors, and an expert in tobacco control research.

**Results:**

Seven themes were extracted from participant interviews, including the type and price of cigarette, the best time to sell cigarettes, profits from the sale of cigarette, affordability, rise in cigarette price and smokers’ reaction to it, lobbying and black-market sales of cigarettes, and the sale and distribution of cigarettes across the country. Although the price of cigarettes in Iran has shown some increases in the past decade, the timing of these increases are not predictable and the limited amount of these increases has not reduced the use of cigarettes. Following a price increase, consumers are more likely to switch from buying packets to single cigarettes, or buy a less expensive brand, then to quit. Moreover, increases in prices may encourage smokers and sellers to buy a large number of cigarettes and store them for a rainy day. Another adverse effect may be increased smuggling of illicit cigarettes to balance the pressure caused by rising prices.

**Conclusions:**

Our findings highlight two important aspects concerning cigarette pricing in Iran. First is the change in the type of purchase from the whole box of cigarettes to the single stick cigarette or swapping to less expensive cigarettes. Second, increase in cigarette price (either through taxing or regular increases) could be offset by flooding smuggled cigarettes into the market. Therefore, in addition to raising cigarette prices, reducing cigarette consumption rates in Iran requires the development and effective implementation of regulatory policies to control cigarette smuggling, reduce purchasing, and subsequently curb the use of this leading cause of premature morbidity and mortality.

**Supplementary Information:**

The online version contains supplementary material available at 10.1186/s12889-021-11733-5.

## Background

Cigarette smoking is a leading cause of preventable morbidity and mortality and is responsible for more than 8 million deaths worldwide annually, mostly from low-and-middle-income countries [1]. According to the World Health Organization (WHO), 10% of Iranians 18 years of age and older smoke cigarettes [2]. Cigarettes are the most popular form of tobacco in Iran and a 2013 meta-analysis of 17 studies revealed that one-fifth of Iranian men and 2–3% of women smoke cigarettes daily [3]. Effective tobacco control policy is needed to combat this problem.

Increasing taxes and prices are effective at reducing overall tobacco consumption [4, 5] by preventing initiation––especially among youth [2] and reducing the level of consumption among established smokers [6], leading to increased tax revenue [7, 8] and long-term reductions in tobacco-related morbidity and mortality [7, 9]. Smokers with low incomes are more responsive to price change than those with higher income [10], and on a population level, economic downturns are associated with decreases in cigarette consumption [11, 12], which shows that affordability may also affect the purchase and consumption of cigarettes in addition to the absolute price of cigarettes.

In Iran, cigarette prices are relatively low compared to neighboring countries such as Turkey and Azerbaijan. Factors in Iran that drive this disparity include the decreased value of currency due to economic sanctions, low taxation of cigarettes, pervasive tobacco marketing, low average quality of local tobacco products, and more importantly smuggling illicit tobacco products which is stimulated by Iran’s weak currency [13]. For example, in 2018, the total tax for a pack of 20 cigarettes of the most popular brand in Iran was 21.7% [2], compared with more than 70% in most European countries [1]. This lack of price harmonization for tobacco products between countries can produce consumption distortions [14]. For instance, when a neighboring country has higher prices, clusters of high sales can be generated in a country with lower prices such as Iran. The opposite is true in the border (the area that a particular governing body controls) of countries when cigarette prices are lower in their neighboring countries. In this situation, the sale of cigarette clusters is lower [14]. As alluded to above, these variations can lead to consumption distortions even if one of the countries implement effective tobacco control policies (e.g., increasing the price). Lower prices in the neighboring country will increase the smuggling of the same tobacco products with lower price and subsequently undermine tobacco control laws. Therefore, it is critical for neighboring countries to have harmonized tobacco control policies under the WHO Framework Convention on Tobacco Control (WHO FCTC) to prevent smuggling and optimize successful tobacco control-related regulations [2].

Iran constitutes one of the biggest Middle East markets targeted by the international tobacco industry [15]. This is compounded by tobacco smuggling activities that supply the market with large quantities of cheap tobacco products without customized warning labels, making cigarettes more affordable for young users, thus stimulating consumption [15].

Indeed, cigarettes have become less affordable between 2008 and 2018 in Iran, making smuggled cigarettes as more prevalent type of cigarettes among smokers [2].

Given Iran’s low tobacco taxes [16], imposing higher taxes could be a useful policy tool to control smoking initiation and intensity [16, 17]. Raising the retail price of tobacco products through increased taxes can reduce consumption, particularly among youth who are in the initial stages of their smoking trajectories and are not yet addicted to nicotine. Furthermore, increasing taxes and prices of tobacco products may be more effective when simultaneous measures are implemented to eliminate illicit trade from all forms of tobacco products [18]. Indeed, curbing illicit tobacco grade will probably be a necessary adjunct to taxation because in response to a drop in cigarette sales following price increases, tobacco companies are likely to implement lobbying efforts, discount schemes, or price-reducing marketing to weaken the impact of tobacco tax increases [16]. A cross-sectional study conducted among 546 smokers reported roughly 20% of tobacco products consumed in Tehran as illicit or smuggled. The same study concluded that raising tobacco prices could decrease consumption without increasing smuggling [19] but it is well established globally that when tobacco taxes increase, cigarette smuggling also increases [20]. Further, foreign brands already are popular in Iran. According to a study that was conducted in Tehran, foreign brands (vs domestic brands) of cigarettes were in high demand, and many smuggled versions of these brands were (or most probably are) available [21]. The key to tackling cigarette smuggling is to cut off supply to the illicit market. This strategy should be at the heart of the WHO FCTC protocol on the illicit trade in tobacco products [22]. According to FCTC “in addition to the damage caused to health, the illicit trade in tobacco is a form of tax evasion and thus also inflicts significant economic harm.” [23].

To date, limited studies have been conducted on the relationship between cigarette prices and their consumption, sale, and distribution in Iran. Therefore, this interview-based qualitative research aimed to identify factors associated with the use, sale, and distribution of cigarettes in Iran, focusing on the relationship between cigarette price and its intake.

## Methods

The present interview-based qualitative study was conducted in Tehran, Iran. Individuals who were cigarette smokers at the time of study or were involved in retail or large-scale cigarette sales, responsible for monitoring and controlling part of the production, distribution, or import of cigarettes in Iran were included. Using purposeful sampling with maximum variation through snowball technique [24], we recruited 20 participants including 12 cigarette smokers, 3 retail shop owners who were selling cigarettes, 4 shop owners who were engaged in large scale distribution of cigarettes in the market in a large scale, and one expert in tobacco research who focused mainly on cigarettes trade and marketing. Prior to each interview, written informed consent was obtained from the participants.

Self-reported data on demographic information (age, marital and employment status, and education) and smoking patterns (age of starting smoking, frequency of smoking) were collected prior to each interview (Table 1). In-depth, semi-structured face-to-face interviews took place from March to July 2016 and lasted between 60-90 min. The interview guide included introductory, probing, and indirect questions. Tobacco consumers were asked initial questions according to interview guide (supplementary file 1) and subsequent questions were asked based on initial responses. Probing questions were used as needed, including “What do you mean?” Or “Can you please explain more.” Participants who were tobacco traders and the tobacco control expert were asked about socio-environmental factors related to cigarette consumption, sale, and distribution of cigarettes, as well as laws related to cigarette pricing, selling and distribution. Sampling continued until data saturation was reached and the most appropriate semantic unit was selected. All participants were offered opportunities to make any extra comments they wished to have noted for their interview. Participants were also assured that their personal information would be kept strictly confidential. Interviews were audio-recorded with the permission of the participants. In the next step, interviews were transcribed verbatim and then checked for accuracy [25].

**Table 1:** Demographic information and smoking pattern of the participants (*n* = 12)

	Frequency/mean	Percent/SD
**Age**	34.4	14.0
**Age of starting smoking**	21.0	10.0
**Marital status**		
Married	4	33
Single	8	67
**Employment situation**		
Employed	10	83
Unemployed	2	17
**Number of cigarettes smoke per day**		
1–10	8	67
11–20	3	25
31–40	1	8
**Education**		
≤ High school	4	33
> High school	8	67

A qualitative content analysis method was used to analyze the obtained data in 3 steps [26]. First, the audio recording of the interviews was typed, and the transcripts were then carefully studied to reach an overall understanding. Second, the MAXQDA10 software was used to organize the interview transcripts by open coding. A review of extracted codes helped to identify their similarities and differences for classification. Finally, with successive analyses, the relationship between specific classes of responses was determined and emerging themes were extracted.

## Results

### Demographic characteristics of participants

Among the 12 cigarette smokers, mean age (± standard deviation) was 34.4 ± 14.0 years and mean age at smoking initiation was 21.0 ± 10.0 years. Demographic characteristics of the participants are presented in Table 1.

As shown in Table 2 and described below, 7 themes emerged in analysis of the qualitative data.

**Table 2:** Categories of the qualitative data

	Categories
1	Type and price of cigarette:
2	Best time to sell cigarettes
3	Profits from the sale of cigarettes
4	Affordability
5	The increase in cigarette price and smokers’ reaction to it
6	Lobbying and black market sales of cigarettes
7	The sale and distribution of cigarettes in the country

### Type and price of cigarette

The median number of cigarettes smoked was 11 cigarettes per day and participants paid an average of 19000 Rial (equal to $ 0.50 at the time of the study) per pack of 20?. Wholesalers mentioned that almost all available cigarette brands are nationally manufactured or packaged. Three vendors explained that the most prevalent brand in their shops is Winston® and others said all brands sold well with no difference between national and international brands. For example, one participant stated “*When people can afford buying international brands they do so, but when they cannot afford it they switch to national cheap cigarettes like Farvardin.*” Concerning volume of cigarette sales, retail vendors stated that they usually sell about 2000 cigarettes per day (100 packs of 20 cigarettes), with Farvardin (an Iranian cigarette brand) as the cheapest brand that costs about 8000 Rial per packet ($ 0.2; at the time of the study) and the most expensive one Winston® that costs 45000 Rial per pack ($ 1.2).

### Best time to sell cigarettes

Vendors reported that cigarette sales fluctuate throughout the month and year. Cigarette sales increase at the beginning of the month when people get their monthly salary, “*most of the cigarettes we sell occur in the first half of the month which is most probably related to the monthly payment that smokers receive*.” Three vendors also cited the first days of the week as the best-selling time for cigarettes, believing that people would finish off their existing cigarettes during the holidays and buy cigarettes on the first day after leaving home. Three vendors also believed that there was a lot of cigarette sales before Nowruz (Iranian new year) because people want to store cigarettes for their Nowruz holiday: *“Because there is a 15-day holiday on Nowruz smokers buy a lot of cigarettes and store them before the beginning of the holiday.”* Another vendor said cold weather and also Muharram (religious month) are two other best-selling times: *“Because of the cold weather and the need for people to warm up with cigarettes, and also in the month of Muharram because of gatherings and street nightlife, people buy and smoke more cigarettes.”* These experiences show that the amount of cigarettes bought by smokers depends not only on their income but also on religious and non-religious holidays.

### Profits from the sale of cigarette

**Fig. 1:**
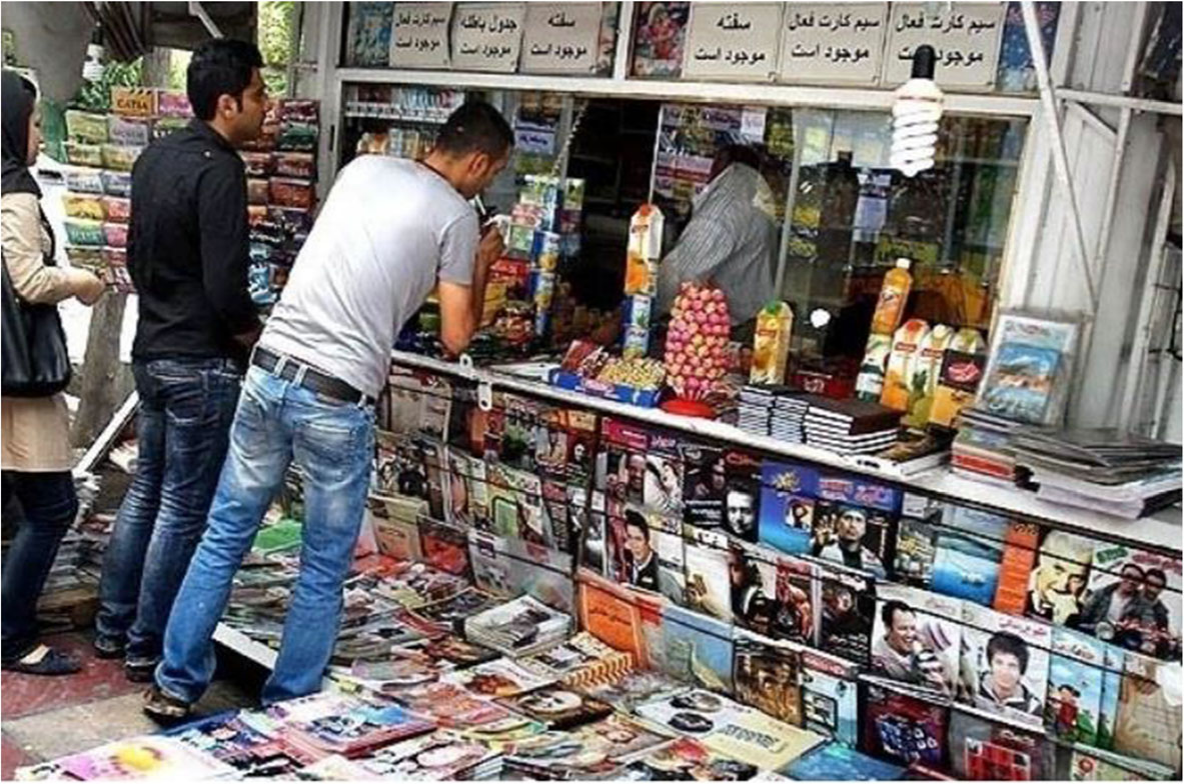
Newsagent’s kiosk in Tehran, Iran. Retrieved from Shafghna News Agent. at https://fa.shafaqna.com/news/582120/

Based on the participants’ experience, the primary source of income for newsagents’ kiosks is cigarette sales (Figure 1; a newsagent kiosk is a small shop selling newspapers and magazines, sweets, and tobacco products). In all kiosks, cigarettes are sold in two forms-- packet or stick. Wholesaler participants believed that retail shops sell fewer cigarettes than wholesale shops, but that profit is greater in retail cigarette sales. They posit that this may be because retail stores can sell single cigarettes, which are more expensive. Two retail and wholesale cigarette vendors stated: “*When you open the box of cigarettes, packets and single cigarettes become more expensive; thus, newsagents’ kiosks’ profits are up to 20%.*” In contrast, another vendor maintained “*profit of cigarette sale differs from brand to brand.*” Kiosks make smoking affordable to young people since they may buy single cigarettes instead of a whole pack of cigarettes.

### Affordability

Cigarette consumers believed that the cost of smoking is very affordable. Nine of the 12 cigarette consumers stated that the price of cigarettes was not much and did not affect their overall living expenses: “*the cost of cigarettes is not much for me and I easily pay for it.*” In general, all 12 cigarette consumers believed that they could easily pay for their cigarettes. However, 9 of them were buying cigarettes very comfortably, and 4 were paying for cigarettes with only a little hardship.

### The increase in cigarette price and smokers’ reaction to it

According to participants, the price of cigarettes all over the country is almost the same. Cigarette prices occasionally increase between 10 and 20 percent across the country but the timing of these increases was not predictable. A wholesaler posited that wholesalers decreased supplies of cigarettes before the Iranian New Year, leading to price increases: “*Before the Nowruz, because of the warehouse, manufacturers do not supply cigarettes to the market and as a result, the price of cigarettes increases.*”

Participants commented that increasing the price of cigarettes does not have a predictable effect on the overall smoking rate: “*I smoke the same amount even if cigarette becomes expensive*"; "*Usually good things become expensive, so if smoking becomes expensive, I am sure it is a good thing, so I smoke more!*” ; *“If cigarettes become expensive even a little, I won’t smoke anymore.”*

Regarding smokers’ reactions to price increases, all vendors told us that when smokers realize the price has gone up, the ones that want to continue to smoke will buy single cigarettes instead of a whole packet: “*When the customer understands the cigarette is expensive, instead of buying a cigarette, they only buy 10 cigarettes*”. Concerning people's emotional responses against the increasing price of cigarettes, two of the retail vendors stated that people think it is our fault and complain about it. One of the vendors said: “*when a brand of cigarette gets expensive, I will not offer that brand in my newsagent’s kiosk for a while, because buyers become upset and angry when they hear the new price. I let them buy their cigarette from elsewhere, and I offer the cigarette when they get used to the new price*.”

### Lobbying and black market sales of cigarettes

Participants were not aware of the legal body or source that determines the cigarette price. Some vendors said that they simply follow the news of cigarette price changes and are unaware of the origin of issuing the law and the news. One of the consumers believed that the parliament increases the price of cigarettes: “*Whenever the parliament approves, cigarette gets expensive*”. Wholesalers stated that Iran Tobacco Company is the only decision-maker of cigarette prices in Iran: *“Apparently, the Tobacco Company makes cigarettes more expensive, but in fact some people lobby and use their influence to determine the price of cigarettes and make a personal profit”.* All participants believed that there are foul players concerning the changes in cigarettes price, which is more pronounced for black market sales’ effect on cigarette prices.

### The sale and distribution of cigarette

This category describes how cigarettes are distributed among retailers and wholesalers when the retail newsagent’s kiosks buy their cigarettes and how this happens. Two of the wholesalers stated that *"Retail newsagents usually buy their cigarette every week."* Another wholesaler mentioned: *"They buy their cigarettes on a daily basis."* He referred to the high rate of newsagent’s kiosks’ sale and stated that “*They would run out of their cigarette supply soon and must buy some more every day.*”

About the distribution of cigarettes throughout the country, five retailers and wholesalers and one tobacco expert believed that there are all kinds of domestic and foreign cigarettes in the market because cigarettes are smuggled into the country by individuals or even governmental lobbies and then distributed throughout the country. According to the person in charge of combating cigarette smuggling in Iran, one of the problems that facilitates the distribution of cigarettes in the country and, consequently, purchase and consumption, is the smuggling of cigarettes. Therefore, to control smoking, one of the necessary measures is to control the smuggling of cigarettes into the country: “*Increasing the price of cigarettes cannot be effective unless the government stops smuggling which provides people with foreign cigarettes at a payable price. In the fight against cigarette smuggling, people cannot easily find illegal cigarettes and subsequently they will reduce their daily consumption.*”

## Discussion

Our findings in this qualitative study provide insights to help decision-makers in setting appropriate pricing policies of cigarettes and control factors associated with cigarette distribution, sale, and consumption in Iran and other developing countries targeted by tobacco industries. According to the results, there is a relationship between the price of cigarettes and the pattern of smoking in Iran. Consistent with previous research [27, 28], our findings showed that the price of cigarettes in Iran is very low and are available in a wide range of prices so that any individual with any level of income can afford to buy cigarettes. Study participants believed that the low price of cigarettes in Iran is indirectly related to the large-scale distribution and accessibility of cigarettes, including smuggled cigarettes.The majority of cigarette vendors referred to Winston® as one of the bestselling cigarettes despite being available in Iran only illicitly. A previous cross-sectional survey among 1540 adult smokers in Iran during 2008–2009 showed that more than 60% of cigarette smokers favored foreign brands [29]. This indicate that foreign cigarettes are supplied on a large scale in Iran, either licit or illicit, and cigarette smokers can easily afford them despite their higher price in the market. Indeed, at the time of writing this report (July 2020), one packet of Winston® in Iran’s market was 60,000 Rials (~$1.4), which increased only by $0.2 since July 2016. It seems that cigarettes prices are not increasing as fast as the drop in the Iranian currency value in the last few years, which in part could explain why cigarette smokers can still afford their preferred brand. Another reason for continuing to smoke in different situations is that smokers can change their brand to pay less but still receive their needed nicotine to suppress withdrawal symptoms and provide positive effects such as increased energy and attention. Indeed, the availability of affordable substitute products is a reason for the high price elasticity of demand for cigarettes [30–32].

The availability of single cigarettes for sale in Iran undercuts price increases lowering demand. Interviews with retail vendors revealed that they make much more profit than wholesalers, and selling single cigarettes makes more profit for them than selling whole packets. A similar study in Guatemala found that the sale of 20 single cigarette sticks makes more profit than selling an entire pack of the same cigarette [33]. Moreover, it is estimated that nearly 75% of all cigarettes are sold as single sticks annually in India [34]. Taken together, the wide availability of single sticks of cigarettes may promote smoking, especially among the young generation who are experimenting with a few cigarettes a day before being hooked on the formidable grip of nicotine and progressing to heavy smoking. More importantly, single cigarette purchasers are unlikely to be exposed to the packet's pictorial and/or textual health warning labels [35], which are one of the main approaches to communicate the potential risks and harms of smoking with smokers and raise awareness.

A US-based study showed that the profitability of single cigarette sales motivates vendors to ignore law prohibiting the sale of single cigarettes to everyone, especially to youth [35]. In our study, the median number of cigarettes smoked daily was 11 cigarettes per day, and participants paid an average of 19,000 Rial (equal to $0.50; at the time of the study). None of the cigarette smokers in our study had any trouble affording their preferred brands. This indicates the affordable price of cigarettes in Iran (either due to low taxation rate or highly consumed smuggled cigarettes) is worrisome and may facilitate earlier initiation among youth who otherwise would be nicotine naïve. Accordingly, there is an urgent need to regulate cigarette prices and eliminate smuggling into the country.

Participants in our study perceived that the price of cigarettes often increased in Iran. Some consumers and vendors of cigarettes believed that smoking prevalence and intensity (ie., quantity and frequency of use) will decrease when prices increase, which is in agreement with the existing body of evidence. Previous studies show that increases in cigarette prices or taxes lead to reduced levels of cigarette consumption [24, 36–40]. In Iran, however, because of the low baseline price, many smokers, even low-income smokers, can easily afford the new prices or switch to purchasing single cigarettes to continue smoking their favorite cigarette brands. Venders who participated in our study also maintained that when cigarette price increases in Iran, many consumers will indeed switch from buying packets to single cigarettes to keep their dependency at bay. This shows that even if an increase in the price of cigarettes negatively affects cigarette consumption, this effect may be temporary.Another main finding of this study is related to the sale and distribution of cigarettes in the country. The wholesalers expressed that the newsagent’s kiosks purchase their cigarettes on a daily or weekly basis. This suggests that the sale of cigarettes in these kiosks is very high and they sell all their cigarette stock on a daily or weekly basis and need to replace it consistently. The primary revenue for the owners of these kiosks is from selling tobacco products either the whole pack or as single sticks of cigarette(s) [41]. This widespread and easy distribution of cigarettes in Iran works against tobacco control efforts.

In our study, participants believed lobbying efforts are behind the illegal import of cigarettes into the country, which in turns lowers the price of cigarettes in the market. Lobbying is a pervasive challenge to regulatory efforts to thwart tobacco smuggling [19, 23]. Although the tobacco industry consistently argues that tax and price increase may lead to contraband [42], an analysis of cigarette prices and levels of smuggling in Iran [19] and sixteen European countries found no correlation between the increase in tobacco prices or taxation and rates of smuggling tobacco activity [42]. However, when it comes to a country under economic sanctions like Iran, illicit cigarettes are significantly less expensive than ones imported legally, making them more affordable to low-income smokers from lower-class families, which poses detrimental health effects on them, their families, and society in general [43]. Additionally, the open sale of illicit cigarettes (e.g., at the newsagent’s kiosks), for which no tax has been paid, is imposing large revenue losses on the Iranian government that otherwise can be spent on the healthcare system [29] or be dedicated to providing smoking cessation services. According to other studies, the economic cycle can also influence cigarette and other illicit drug consumption and also the mental and physical health of the population [44, 45]. Additionally, high price elasticity of demand for cigarettes could increase tax evasion [46]. Future observational and experimental studies are needed to better understand the aforementioned challenges and propose effective regulatory solutions.

This study has some limitations. Conducting a study solely on consumers and vendors in Tehran reduces the generalizability of the results. However, we performed in-depth interviews with a variety of participants (ranging from venders to consumers and an expert in tobacco research) and tried to reach a deep understanding of the relationship between cigarette price and its consumption. Due to the nature of our study, we didn’t divide participants into various income or occupation groups when we asked them about the effect of cigarette prices on the rate of their cigarette consumption. Therefore, we cannot conclude which income or occupation groups are more sensitive to cigarette price increases; however, existing evidence indicates some socially deprived smokers will reduce essential household spending to maintain smoking on a budget that is already limited [47]. Therefore, even poor smokers may continue buying and using cigarettes by the mechanism explained above. At the level of cigarette retailers, major vendors sometimes do not trust researchers and this may make them to give short or incomplete responses to some questions.Despite these limitations, our study has important implications for policy and research. Based on our findings, we suggest several steps to help guide policymakers in Iran to control the consumption of cigarette, including 1) substantially increasing price through enhanced taxation, 2) enhancing enforcement and control on contraband sales of cigarette, and 3) enhancing prevention campaigns activities (school- and family-based), especially preventing youth from initiating cigarette smoking, 4) banning cigarette sale in newsagent’s kiosks, 5) banning or enforcing the ban on selling single sticks of cigarettes, and finally, 6) Requiring complementary strategies including smoking cessation services, tobacco control campaigns to prevent smoking initiation among youth, and strict enforcement of laws limiting access of minors to tobacco products.

## Conclusion

Our findings demonstrated that the cost of cigarettes in Iran is low for consumers. Although sometimes the price of cigarettes in Iran increases, neither the time of this price increase nor its amount is such that it reduces smoking. When cigarettes become more expensive, consumers who cannot afford the price increase adapt their behavior to maintain their smoking habit by switching from purchasing packets to single cigarettes or switching to a less expensive brand. The fact that the newsagent’s kiosks purchase their cigarettes daily or weekly shows the high rate of consumption and the widespread and easy distribution of cigarettes in Iran. As such, any upsurge in the price of cigarettes in Iran will not lead to a decrease in purchases and consumption because it will soon be offset by an increase in the distribution of cigarettes. This may cause more smuggled cigarettes to enter the country as well. Taken together, we conclude that in addition to increasing prices and taxes, reducing rates of cigarette consumption in Iran requires the development and effective implementation of regulatory actions to curb cigarette smuggling and the sales of single sticks of cigarettes. Building national capacity to develop (and implement) an effective and sustainable national tobacco control program is an urgent priority in Iran and this should include monitoring of cigarette prices and sales over time to deliver on-time and accurate information to guide tobacco control regulatory policies.

## Supplementary Information

**ESM 1.**

## Data Availability

The datasets generated during and analyzed during the current study are not publicly available due to privacy protection issues but are available from the corresponding author on reasonable request.

## References

[1] World Health Organization. WHO report on the global tobacco epidemic 2019: Offer help to quit tobacco use 2019.

[2] WHO report on the global tobacco epidemic, 2019 Country profile Iran (Islamic Republic of). Available at https://www.who.int/tobacco/surveillance/policy/country_profile/irn.pdf

[3] Moosazadeh M, Ziaaddini H, Mirzazadeh A, Ashrafi-Asgarabad A, Haghdoost AA (2013). Meta-analysis of smoking prevalence in Iran. Addiction & health.

[4] Ross H, Stoklosa M, Krasovsky K (2012). Economic and public health impact of 2007–2010 tobacco tax increases in Ukraine. Tob Control.

[5] Chaloupka FJ, Straif K, Leon ME (2011). Effectiveness of tax and price policies in tobacco control. Tob Control.

[6] de Miera Juárez BS, Thrasher JF, Shigematsu LMR, Ávila MH, Chaloupka FJ (2014). Tax, price and cigarette brand preferences: a longitudinal study of adult smokers from the ITC Mexico survey. Tob Control.

[7] Nargis N, Ruthbah UH, Hussain AG, Fong GT, Huq I, Ashiquzzaman S (2014). The price sensitivity of cigarette consumption in Bangladesh: evidence from the international tobacco control (ITC) Bangladesh wave 1 (2009) and wave 2 (2010) surveys. Tob Control.

[8] Ross H, Al-Sadat NA (2007). Demand analysis of tobacco consumption in Malaysia. Nicotine Tob Res.

[9] Ross H, Chaloupka F (2006). Economic policies for tobacco control in developing countries salud pública de méxico.

[10] Lewis S, Arnott D, Godfrey C, Britton J (2005). Public health measures to reduce smoking prevalence in the UK: how many lives could be saved?. Tob Control.

[11] Almeida A, Golpe AA, Iglesias J, Martín Álvarez JM (2021). The Price elasticity of cigarettes: new evidence from Spanish regions, 2002–2016. Nicotine and Tobacco Research.

[12] Álvarez JMM (2020). Price and income elasticities of demand for cigarette consumption: what is the association of price and economic activity with cigarette consumption in Spain from 1957 to 2016?. Public Health.

[13] Batmanghelidj E, Heydari G (2014). Sanctions, smuggling, and the cigarette: the granting of Iran office of foreign asset control's licenses to big tobacco. Int J Prev Med.

[14] Almeida A, Golpe AA, Martín Álvarez JM (2020). A spatial analysis of the Spanish tobacco consumption distribution: are there any consumption clusters?. Public Health.

[15] World Health Organization, Research for International Tobacco Control. WHO report on the global tobacco epidemic, 2008: the MPOWER package: World Health Organization; 2008.

[16] Raei, Behzad, et al. "Impact of simulated cigarette excise tax increase on its consumption in Iran." Epidemiology and Health 42 (2020): e2020054. 10.4178/epih.e2020054 PMC7871162 32777885

[17] Rad H, Enayatollah, et al. (2021). Quality and quantity of price elasticity of cigarette in Iran. Int J Health Plann Manag.

[18] Poorolajal J, Mohammadi Y, Mahmoodi A. Challenges of tobacco control program in Iran. Archives of Iranian Medicine. 2017;20(4):0-. 28412827

[19] Abdollahpour I, Mansournia MA, Salimi Y, Nedjat S (2019). Lifetime prevalence and correlates of smoking behavior in Iranian adults’ population; a cross-sectional study. BMC Public Health.

[20] Yürekli A, Sayginsoy Ö (2010). Worldwide organized cigarette smuggling: an empirical analysis. Appl Econ.

[21] Heydari G, Tafti SF, Telischi F, Joossens L, Hosseini M, Masjedi M, Ghafari M (2010). Prevalence of smuggled and foreign cigarette use in Tehran, 2009. Tob Control.

[22] Joossens L, Raw M (2008). Progress in combating cigarette smuggling: controlling the supply chain. Tob Control.

[23] https://www.who.int/fctc/publications/The_TI_and_the_Illicit_Trade_in_Tobacco_Products.pdf?ua=1

[24] Palinkas LA, Horwitz SM, Green CA, Wisdom JP, Duan N, Hoagwood K (2015). Purposeful sampling for qualitative data collection and analysis in mixed method implementation research. Adm Policy Ment Health Ment Health Serv Res.

[25] Hoek J, Smith K (2016). A qualitative analysis of low income smokers’ responses to tobacco excise tax increases. Int J Drug Policy.

[26] Hsieh H-F, Shannon SE (2005). Three approaches to qualitative content analysis. Qual Health Res.

[27] Ekpu VU, Brown AK. The economic impact of smoking and of reducing smoking prevalence: review of evidence. Tobacco use insights. 2015;8:TUI. S15628. 10.4137/TUI.S15628 PMC4502793 26242225

[28] He Y, Shang C, Chaloupka FJ (2018). The association between cigarette affordability and consumption: an update. PLoS One.

[29] Heydari G, Joossens L, Chamyani F, Masjedi MR, Shadmehr MB, Fadaizadeh L (2016). Second pack survey on the prevalence of the use of smuggled cigarettes in Tehran, 2015. Tob Control.

[30] Kinh V, Hoang, et al. (2006). The effect of imposing a higher, uniform tobacco tax in Vietnam. Health Research Policy and Systems.

[31] Gligorić D, Pepić A, Petković S, Ateljević J, Vukojević B (2020). Price elasticity of demand for cigarettes in Bosnia and Herzegovina: microdata analysis. Tob Control.

[32] Selvaraj, Sakthivel, Swati Srivastava, and Anup Karan. "Price elasticity of tobacco products among economic classes in India, 2011–2012." BMJ open 5.12 (2015). 10.1136/bmjopen-2015-008180 PMC4679943 26656009

[33] de Ojeda A, Barnoya J, Thrasher JF (2012). Availability and costs of single cigarettes in Guatemala. Nicotine Tob Res.

[34] Lal P, Kumar R, Ray S, Sharma N, Bhattarcharya B, Mishra D, Sinha MK, Christian A, Rathinam A, Singh G (2015). The single cigarette economy in India-a Back of the envelope survey to estimate its magnitude. Asian Pac J Cancer Prev.

[35] Latkin CA, Murray LI, Smith KC, Cohen JE, Knowlton AR (2013). The prevalence and correlates of single cigarette selling among urban disadvantaged drug users in Baltimore, Maryland. Drug Alcohol Depend.

[36] Cetin T (2017). The effect of taxation and regulation on cigarette smoking: fresh evidence from Turkey. Health Policy.

[37] Hammamizade O, Mazaheri Tehrani A, Hajiketabi S, Khatami S, Fathi Moghadam M, Rahimi H (2015). Smoking frequency and some related factors among high school students of Kashan City. Iran International Archives of Health Sciences.

[38] Choi SE (2016). Are lower income smokers more price sensitive?: the evidence from Korean cigarette tax increases. Tob Control.

[39] Guindon GE, Paraje GR, Chaloupka FJ (2015). The impact of prices and taxes on the use of tobacco products in Latin America and the Caribbean. Am J Public Health.

[40] Jha P, Chaloupka FJ (2000). The economics of global tobacco control. Bmj..

[41] Tasnim news agency. Newspaper or Cigarette Stalls? / Super Luxury Stalls with Astronomical Prices 3. 2019.

[42] Joossens L, Raw M (1998). Cigarette smuggling in Europe: who really benefits?. Tob Control.

[43] Arevalo R, Corral JE, Monzon D, Yoon M, Barnoya J (2016). Characteristics of illegal and legal cigarette packs sold in Guatemala. Glob Health.

[44] Álvarez, Juan M. Martín, et al. (2020). Asymmetric behavior of tobacco consumption in Spain across the business cycle: a long-term regional analysis. International Journal of Health Economics and Management.

[45] Bellés-Obrero, Cristina, and Judit Vall Castelló. "The business cycle and health." Oxford Research Encyclopedia of Economics and Finance. 2018.

[46] Chaloupka, Frank J., and John A. Tauras. "The demand for cigarettes in Ireland." Health Service Executive’s National Tobacco Control Office (2011).

[47] Guillaumier A, Bonevski B, Paul C (2015). ‘Cigarettes are priority’: a qualitative study of how Australian socioeconomically disadvantaged smokers respond to rising cigarette prices. Health Educ Res.

