# Supplementary material for: The effect of price on cigarette consumption, distribution, and sale in Tehran: a qualitative study
Source: BMC Public Health. 2021 Sep 22;21:1720. doi: 10.1186/s12889-021-11733-5 (PMC8456583; doi:10.1186/s12889-021-11733-5)
Supplement: Supplementary file 1 — ESM 1. [file 12889_2021_11733_MOESM1_ESM.pdf]

**The interview guide:**

**Questions from tobacco consumers:**

1. How did you start smoking?
2. Where do you buy your cigarette from?
3. How is the price of cigarette for you?
4. If the price of cigarette changes, do you continue smoking?

**Questions from cigarette traders and the expert of combating cigarette smuggling:**

1.What do you think about socio-environmental factors related to cigarette consumption, sale and distribution of cigarette?

2. What do you think about the laws related to cigarette pricing, selling and distribution?

**Probing questions including wherever needed:**

1. What do you mean?
2. Can you please explain more?
